# Identification and validation of oxeiptosis-associated lncRNAs and prognosis-related signature genes to predict the immune status in uterine corpus endometrial carcinoma

**DOI:** 10.18632/aging.204726

**Published:** 2023-05-19

**Authors:** Linjun Niu, Zhengyuan Wu

**Affiliations:** 1 Department of Hand Plastic Surgery, The First People’s Hospital of Linping District, Hangzhou 311199, China; 2 Department of Oncology, Huaibei People’s Hospital, Huaibei 235000, China

**Keywords:** uterine corpus endometrial carcinoma, oxeiptosis, lncRNA, risk signature, prognosis, immune status

## Abstract

As a novel cell death modality, oxeiptosis is mainly caused by oxidative stress. However, the associations of uterine corpus endometrial carcinoma (UCEC) with oxeiptosis-associated long non-coding RNAs (lncRNAs) are unknown. Here, to identify hub oxeiptosis-associated lncRNAs in UCEC, we collected the data for lncRNAs and gene expression in UCEC from The Cancer Genome Atlas (TCGA) database. Then, a lncRNA risk signature was constructed, and its prognostic value was further evaluated. Finally, the expression levels of hub lncRNA *HOXB-AS3* were validated by quantitative RT-PCR analysis. MTT and wounding analyses were also applied to confirm the role of HOXB-AS3 knockdown on UCEC cells. Five lncRNAs associated with oxeiptosis and connected to the prognosis of UCEC were identified, and a risk signature was constructed based on these identified lncRNAs. Our clinical value analyses suggested that the risk signature was closely connected to the overall survival, TNM stage, and grade of UCEC patients. Meanwhile, compared to the conventional clinicopathological characteristics, this risk signature exhibited significantly higher diagnostic accuracy. Moreover, the potential mechanism analysis indicated a close connection of this risk signature to tumor stemness, m6A-related genes, immune cell infiltration, and immune subtypes. Based on the risk scores, we constructed a nomogram. *In vitro* experiments found that *HOXB-AS3* was significantly higher expressed in UCEC cells, and the silence of *HOXB-AS3* inhibited the proliferation and migration of UCEC cells. In conclusion, using five hub lncRNAs associated with oxeiptosis, we generated a risk signature, which could be applied in the novel therapeutic strategies of UCEC development.

## INTRODUCTION

As the main cause of death in female tumor patients, uterine corpus endometrial carcinoma (UCEC) results 12,550 and 17,543 deaths in United State and China women, respectively, according to the 2022 cancer statistics [1, 2]. It is regarded as a frequent gynecological malignant tumor with high mortality and seriously threatens public health. Despite surgery, various neoadjuvant therapies, such as radiotherapy, chemotherapy, and immunotherapy, have also been employed to UCEC treatment recently. However, their curative effect is still controversial, and numbers of UCEC patients remain dismal prognosis [3]. Therefore, screening and identifying novel therapeutic targets and the construction of sensitive prognostic signatures that can accurately predict a patient’s condition is urgently needed.

According to the triggering mechanism, the cell death program primarily follows two paths: programmed cell death (PCD) and accident cell death (ACD) [4]. As an apoptosis-like, caspase-independent cell death modality, oxeiptosis was recently identified as a novel PCD mechanism that is significantly connected with the pathological accumulation of reactive oxygen species (ROS) [5]. Convincing data has defined that the *KEAP1* (Kelch-like ECH-associated protein 1)-*PGAM5* (Phosphoglycerate mutase family member 5)-*AIFM1* (Apoptosis-inducing factor mitochondria-associated 1) pathway is significantly involved in the regulation of oxeiptosis [6]. As a virtual sensor for reactive oxygen, *KEAP1* can steadily increase the level of *Nrf2* [7] and then enhance several anti-oxidation-related genes expression, thus protecting the cells against moderate ROS concentration stress [8]. In high concentrations of intracellular ROS, oxeiptosis can utilize the ROS sensing capabilities of *KEAP1* to induce a PCD [5]. In such conditions, *KEAP1* will disassociate from PGAM5, subsequently internalize PGAM5 into the lumen of mitochondria, and finally promote *AIFM1* dephosphorylation [9]. Consequently, *AIFM1* can be shuttled to the nucleus and promote DNA degradation, including apoptosis and parthanatos, to induce chromatin condensation [10]. Recently, a study has also proved that oxeiptosis is significantly involved in the prognosis of breast cancer [11]. Nevertheless, the specific role of oxeiptosis in UCEC is still unclear.

As a type of non-coding RNA, long non-coding RNAs (lncRNAs) significantly participate in the process of tumor progression, such as tumor cell growth, tumorigenesis, and metastasis [12, 13]. Additionally, dramatic associations of lncRNAs with the overall survival (OS) of cancer patients have also been observed [14]. The regulation of lncRNAs in cancer cell migration, invasion, apoptosis, and cell cycle progression has also been demonstrated by several *in vitro* and *in vivo* experimental studies. Therefore, lncRNAs are considered a critical factor in UCEC prognosis. However, the relationship of oxeiptosis-associated lncRNAs with UCEC prognosis has not been explored.

Along with the development and application of bioinformatic analysis, researchers have identified many disease-specific biomarkers. However, no lncRNAs associated with oxeiptosis and UCEC prognosis or progression have been identified so far. Therefore, using univariate Cox regression and gene expression analyses, we screened the lncRNAs which significantly associated with UCEC patient prognosis and also differentially expressed between normal and UCEC patients. Then, after characterizing hub oxeiptosis-associated lncRNAs, we conducted a LASSO penalized Cox regression analysis and established a risk signature. The clinical significance and prognostic value of this risk signature was validated in this study. The connections of this risk signature to tumor stemness, m6A genes, and immune infiltration were also investigated. Moreover, several *in vitro* experiments were constructed to explore the role of hub lncRNA *HOXB-AS3* in UCEC cells. In summary, we first constructed a risk signature using oxeiptosis-associated lncRNAs and provided a useful tool for predicting UCEC prognosis and novel insights for its diagnosis.

## RESULTS

### Screening of prognostic lncRNA candidates

**Figure 1:**
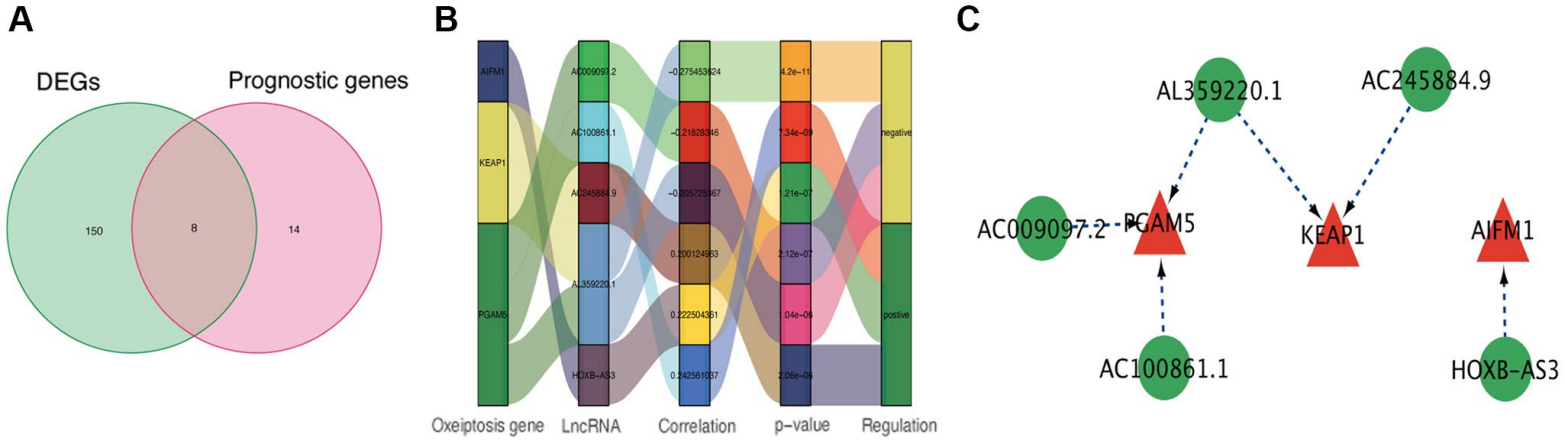
**Identification of prognostic oxeiptosis-associated lncRNAs.** (**A**) Venn diagram of candidate oxeiptosis-associated lncRNAs determined by differential expression and univariate Cox analyses. (**B**) Correlation network of prognostic lncRNAs and their associated mRNAs. (**C**) Correlation network of hub lncRNAs.

Through the correlation analysis with |R^2^| > 0.2 and *p* < 0.05, 723 lncRNAs significantly associated with oxeiptosis genes were identified (Supplementary Table 1) and used for subsequent explorations. Meanwhile, we identified 158 differentially expressed lncRNAs (Supplementary Table 2) and 22 prognosis-associated lncRNAs (Supplementary Table 3) using differential expression and univariate Cox regression analyses. Finally, 8 overlapping lncRNAs were selected as candidate lncRNAs for further prognostic analysis (Figure 1A).

### Risk signature construction

Using the above-identified candidate lncRNAs, we conducted Lasso penalized Cox regression analysis and constructed a risk signature with 5 hub lncRNAs: *HOXB-AS3*, *AC009097.2*, *AL359220.1*, *AC100861.1*, *AC245884.9* (Supplementary Table 4). The connections of these identified hub lncRNAs to the genes related to oxeiptosis are presented in Figure 1B, 1C.

### Prognostic value analysis of oxeiptosis-associated lncRNAs in UCEC

**Figure 2:**
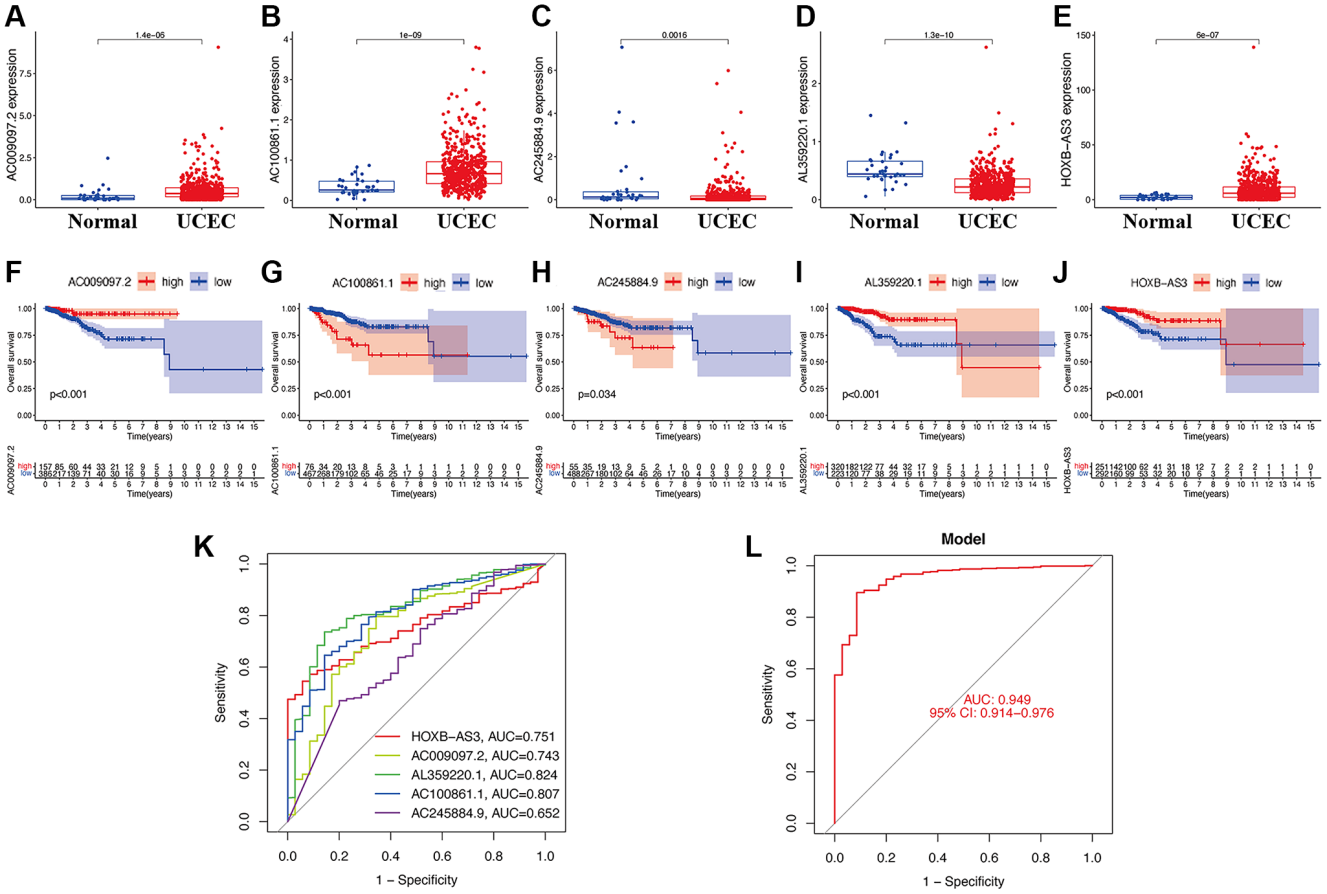
**Clinical value of hub lncRNAs in UCEC.** Gene expression levels of AC009097.2 (**A**), AC100861.1 (**B**), AC245884.9 (**C**), AL359220.1 (**D**), HOXB-AS3 (**E**) in risk subgroups. Survival curve of AC009097.2 (**F**), AC100861.1 (**G**), AC245884.9 (**H**), AL359220.1 (**I**), HOXB-AS3 (**J**) in UCEC. ROC curves of single diagnostic biomarkers (**K**) and prediction model (**L**).

When evaluating the gene expression levels of hub lncRNAs in UCEC, the results show that *AC009097.2* (Figure 2A), *AC100861.1* (Figure 2B), and *HOXB-AS3* (Figure 2E) were significantly higher expressed in UCEC tissues compared to normal samples (*p* < 0.05). In contrast, the others, including *AC245884.9* (Figure 2C) and *AL359220.1* (Figure 2D), were expressed at significantly lower levels in UCEC tissues (*p* < 0.05). The KM survival analysis was further applied to estimate the correlation between lncRNA expression and UCEC prognosis. The results indicated that the higher *AC009097.2* (Figure 2F), *AL359220.1* (Figure 2I), and *HOXB-AS3* (Figure 2J) expression subgroups had a significantly higher OS of UCEC patients (*p* < 0.05). On the other hand, the higher expression of *AC100861.1* (Figure 2G) and *AC245884.9* (Figure 2H) were dramatically connected to the poor prognosis in UCEC patients (*p* < 0.05). As a single diagnostic biomarker, the ROC analysis revealed that the area under the ROC curve (AUC) of *HOXB-AS3* was 0.751, *AC009097.2* was 0.743, *AL359220.1* was 0.824, *AC100861.1* was 0.807, and *AC245884.9* was 652, indicating that all identified lncRNAs had a high predictive accuracy in UCEC patients (Figure 2K). Meanwhile, when all hub lncRNAs were combined into a prediction model, the ROC analysis showed that the predictive accuracy of UCEC increased to 0.949 (Figure 2L).

### Risk signature value in clinics

**Figure 3:**
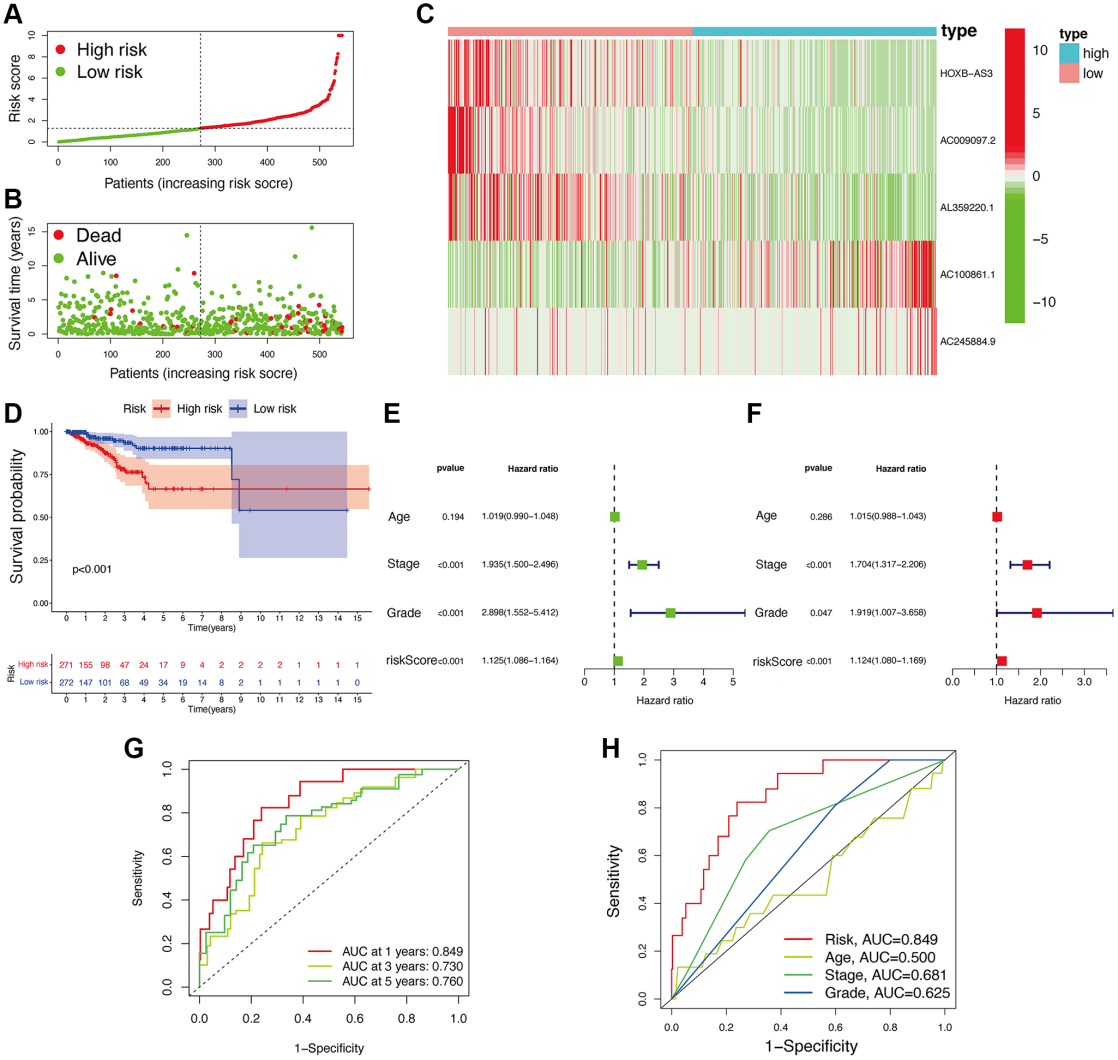
**Associations between risk signature and UCEC prognosis.** Risk score distribution (**A**) and survival status (**B**) analysis of TCGA-UCEC cohort. (**C**) Expression level of hub lncRNAs in risk subgroups. (**D**) Survival curve of UCEC patients. Univariate (**E**) and multivariate Cox (**F**) regression of clinicopathological features. TimeROC (**G**) and ClinicalROC (**H**) curves to forecast overall survival of patients.

Based on the median value of calculated risk scores, we categorized UCEC patients into two subgroups with low- and high-risk scores (Figure 3A, 3B). While evaluating the expression level of lncRNAs in two risk subgroups, Figure 3C disclosed that *AC100861.1* and *AC245884.9* were expressed at significantly higher levels in high-risk subgroup (*p* < 0.05). In contrast, the others, including *AC009097.2, AL359220.1*, and *HOXB-AS3*, were all significantly lower expressed in high-risk subgroup compared to low-risk subgroup (*p* < 0.05). The UCEC patients with high-risk scores showed a significantly lower OS than those with low-risk scores (*p* < 0.05; Figure 3D). Univariate Cox regression analysis also confirmed that the risk signature was significantly connected with OS of UCEC patients (Figure 3E). Additionally, Figure 3F also demonstrated that this risk signature could be used as an independent factor for predicting UCEC patients. A high predictive accuracy of this risk signature at 1 (AUC = 0.849), 3 (AUC = 0.730), and 5 (AUC = 0.760) years were found using ROC curve analysis (Figure 3G). Compared with other traditional clinicopathological features (including age, TNM stage, and cancer grade), a significantly higher accuracy of this risk signature was observed by ROC curve analyses at 1 year (Figure 3H), demonstrating the sensitivity and specificity of this risk signature for OS prediction of UCEC.

**Figure 4:**
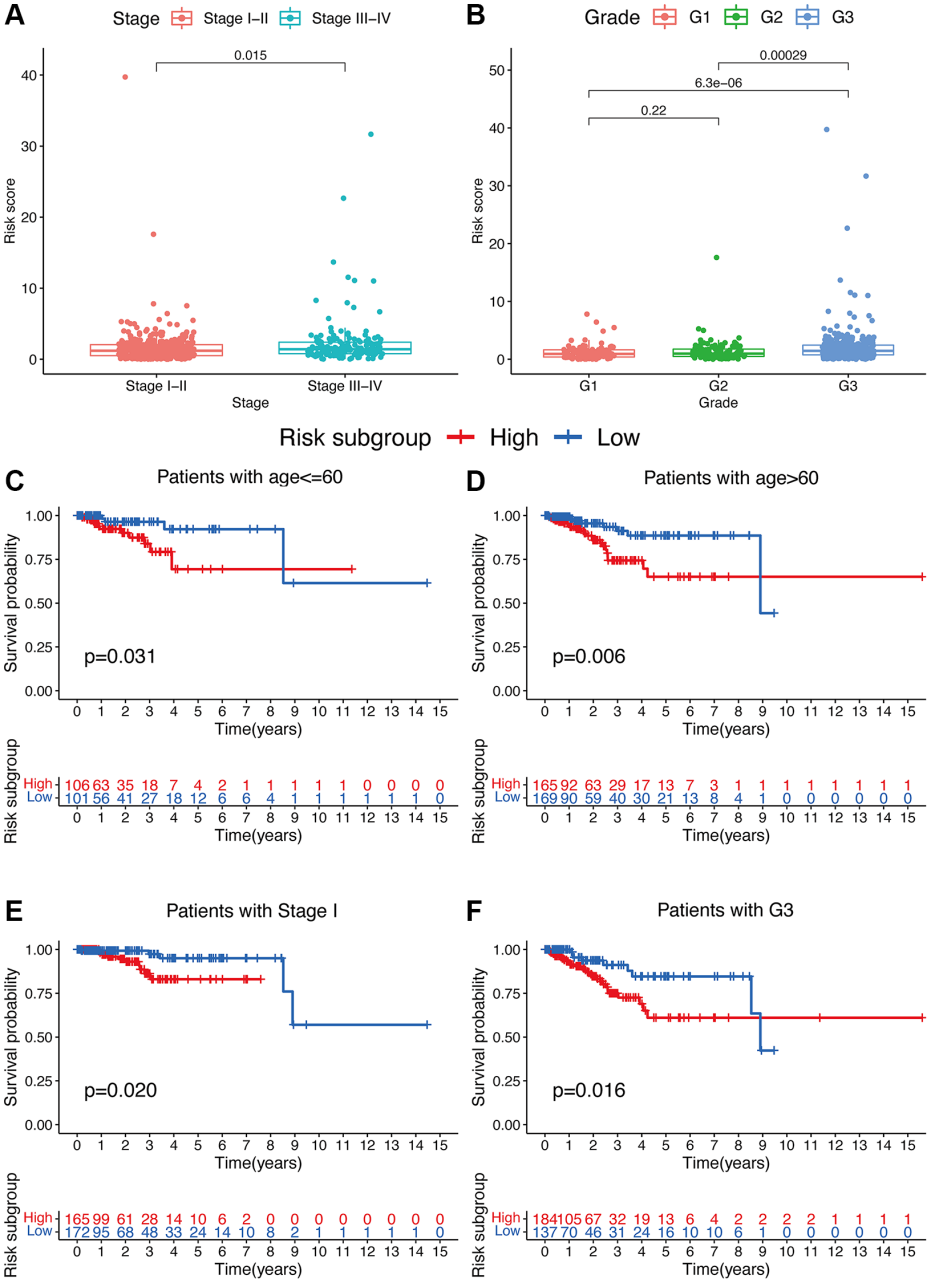
**Associations between risk signature and clinicopathological factors.** Correlations between risk scores and TNM stage (**A**) and grade (**B**). The prognosis of risk signature under the stratifications of (**C**, **D**) age ≤60 and age >60; (**E**) TNM stage I; and (**F**) grade 3.

**Figure 5:**
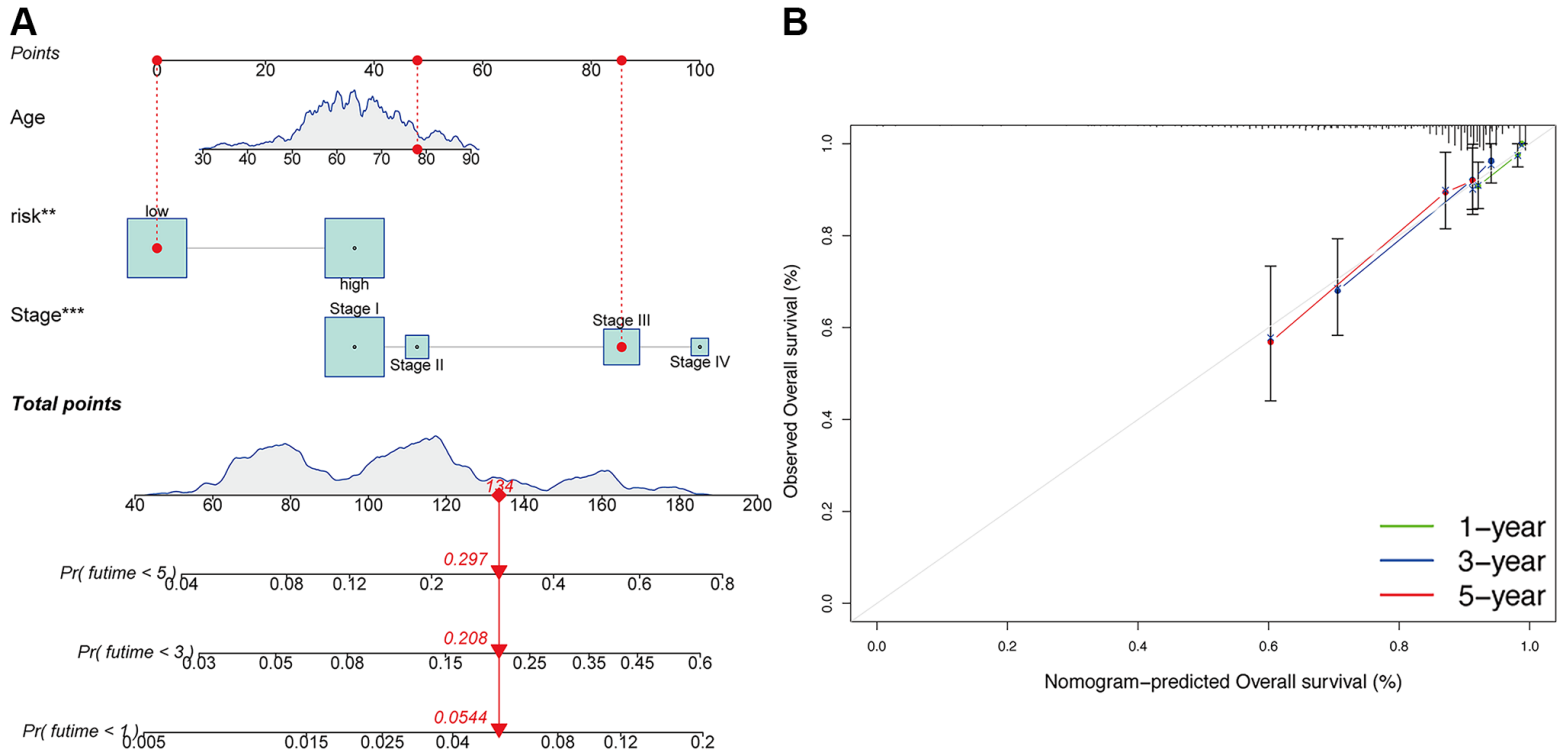
**Construction of nomogram.** (**A**) Nomogram for predicting UCEC 1-, 3-, and 5-year overall survival. The red dashed line represented a sample of UCEC patient's death probability by year 1, 3, and 5. (**B**) Decision curve analysis of risk signature.

Additionally, compared to patients with TNM stage III-IV, patients with stage I-II exhibited significantly lower risk scores (*p* < 0.05; Figure 4A). Meanwhile, compared to patients with grade 1 or 2, obviously higher risk scores were found in patients with grade 3 (*p* < 0.05; Figure 4B). Furthermore, the risk signature’s value for prognosis in UCEC patients with diverse clinical features was investigated. As a result, it was revealed that there existed critical significant differences among low- and high-risk subgroups in patients younger than 60 years old (Figure 4C), over 60 years old (Figure 4D), patients with TNM stage I (Figure 4E), and patients with grade 3 (Figure 4F). In these two subgroups, all high-risk signatures displayed a significant OS disadvantage when compared with the low-risk signature. Finally, to predict the outcome of UCEC patients, we constructed a nomogram using this identified risk signature (Figure 5A), and the calibration curves at 1-, 3-, and 5-year follow-up showed that our nomogram had a substantial agreement (Figure 5B). Overall, due to the close association of the risk signature with UCEC development, our established risk signature might be a valuable tool for managing UCEC patients in clinics.

### Functional enrichment analyses

**Figure 6:**
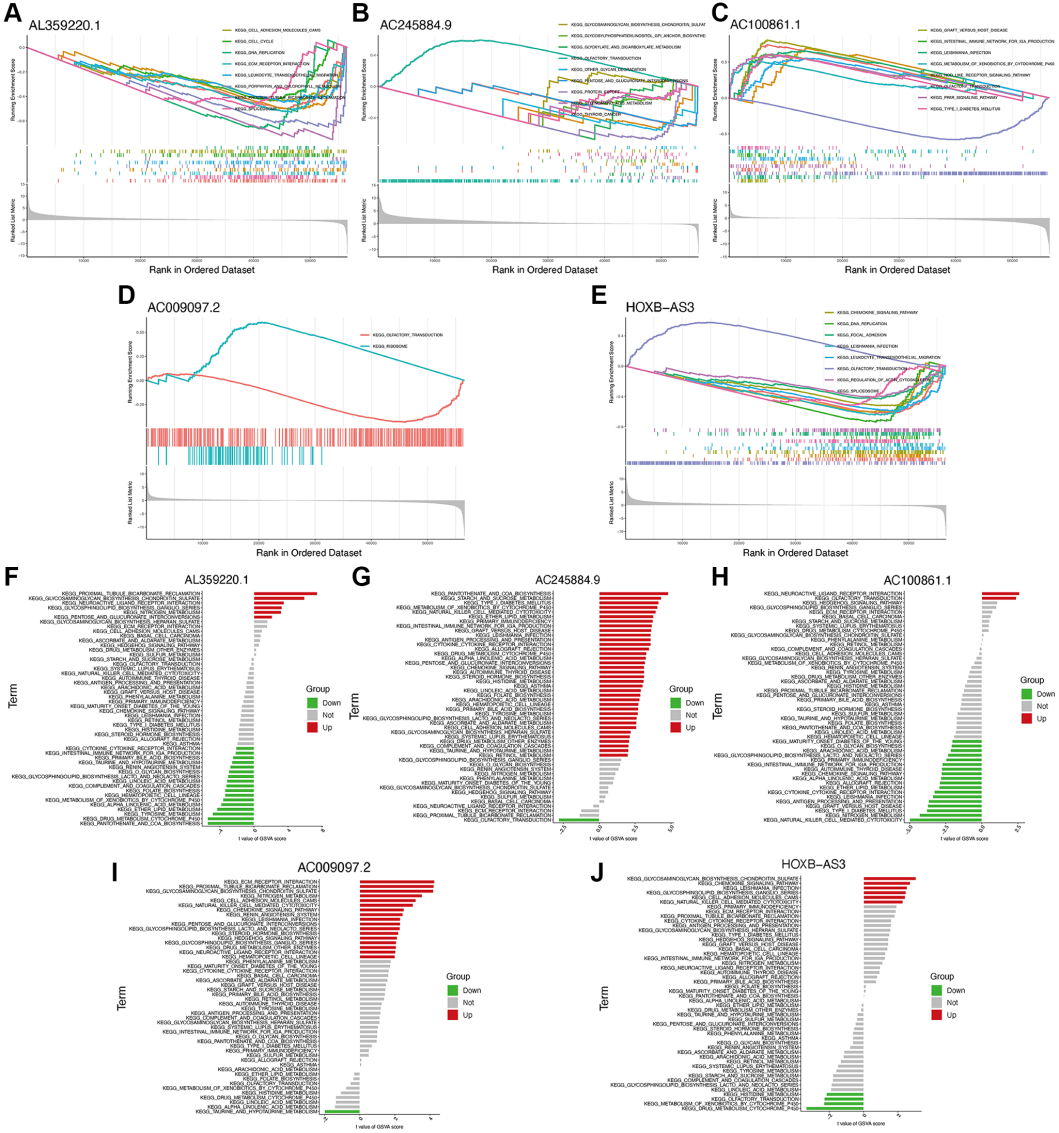
**Functional enrichment analysis of hub lncRNAs.** GSEA analysis of lncRNAs AL359220.1 (**A**), AC245884.9 (**B**), AC100861.1 (**C**), AC009097.2 (**D**), HOXB-AS3 (**E**). GSVA analysis of lncRNAs AL359220.1 (**F**), AC245884.9 (**G**), AC100861.1 (**H**), AC009097.2 (**I**), HOXB-AS3 (**J**).

Using GSEA and GSVA analyses, significant enrichments of the hub identified lncRNAs were identified in pathways such as cell adhesion molecules, cell cycle, and chemokine signaling (Figure 6A–6J). Meanwhile, several immune-related pathways were also enriched, such as primary immunodeficiency, natural killer cell-mediated cytotoxicity, antigen processing and presentation, and immune network for IgA production.

### Immune infiltration of Hub lncRNAs

**Figure 7:**
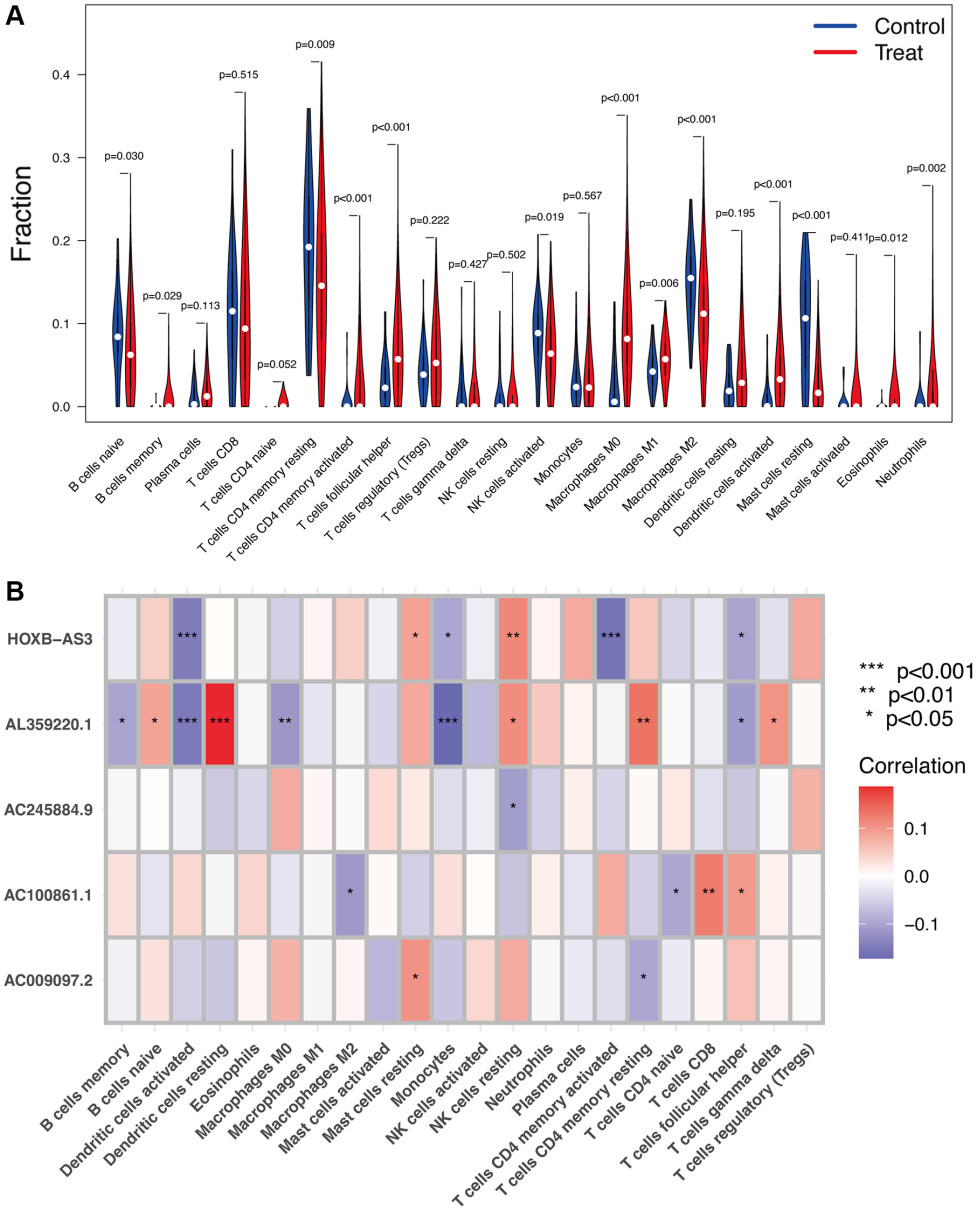
**Immune infiltration analysis.** (**A**) The proportion of 22 types of immune cells between normal control and UCEC samples. (**B**) Correlation heatmap depicting correlations between infiltrated immune cells and hub lncRNAs in UCEC.

As shown in Figure 7A, several immune cells were significantly differential infiltrated between UCEC and control samples by the CIBERSORT algorithm analysis. The results indicated that memory B cells, activated CD4 memory T cells, helper follicular T cells, M0 and M1 macrophages, activated dendritic cells, eosinophils, and neutrophils were significantly upregulated in UCEC samples; however, the proportions of naïve B cells, resting CD4 memory T cells, activated NK cells, M2 macrophages, and resting mast cells, were significantly increased in control samples. The correlation between hub identified lncRNAs and immune infiltration in UCEC was determined. Figure 7B shows that *HOXB-AS3, AC009097.2, AL359220.1, AC100861.1, AC245884.9* were all strongly associated with the content of immune cells, indicating that all these lncRNAs may be prognostic targets for UCEC immunotherapy.

### Relationship of the risk signature with immunity, tumor stemness, and m6A-related genes

**Figure 8:**
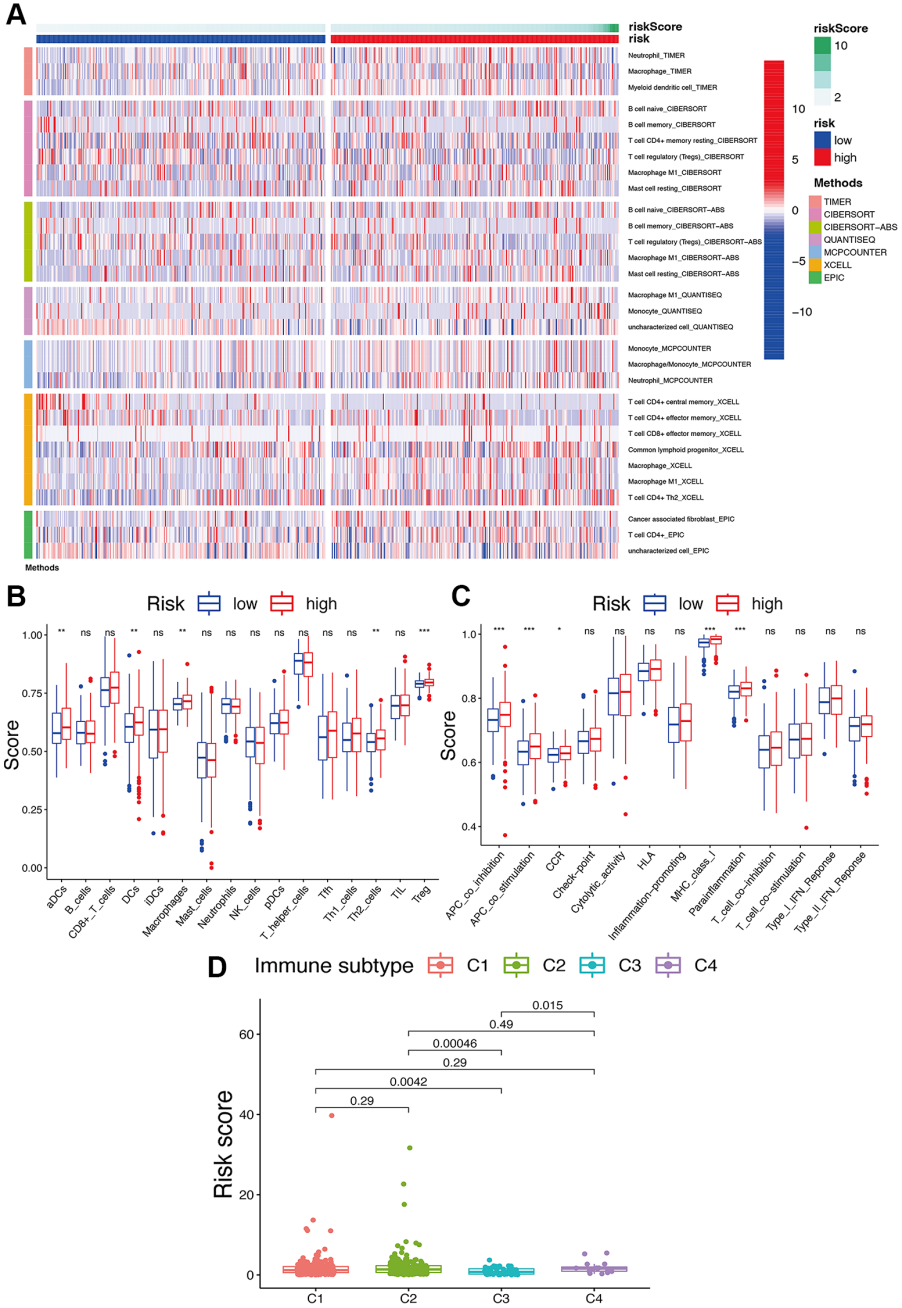
**Potential role of risk signature in UCEC immune status.** (**A**) Heatmap for immune responses based on EPIC, XCELL, MCP counter, QUANTISEQ, CIBERSORT, and TIMER among two risk subgroups. Boxplots of scores of immune cells (**B**) and immune-associated functions (**C**) in risk subgroups. Associations between risk signature and immune infiltration subtypes (**D**).

Based on the TIMER, CIBERSORT, QUANTISEQ, MCP counter, XCELL, and EPIC analyses, a close connection of the risk signature to several immune cells was detected (Figure 8A). A significant reduction of various immune cell subpopulations and functions, including aDCs, DCs, macrophages, Th2 cells, Treg, APC co-inhibition, APC co-stimulation, CCR, MHC class I, and parainflammation, was found in the patients with low-risk scores compared to those with high-risk scores (Figure 8B, 8C) (*p* < 0.05). To further clarify the connections of the risk signature to immune components, we evaluated the immune infiltrates associated with the promotion and suppression of tumors [15], such as wound healing (C1), INF-g dominant (C2), inflammatory (C3), and lymphocyte-depleted (C4) subtypes. Among these subtypes, a significantly lower risk score was found for the C3 subtype (Figure 8D).

**Figure 9:**
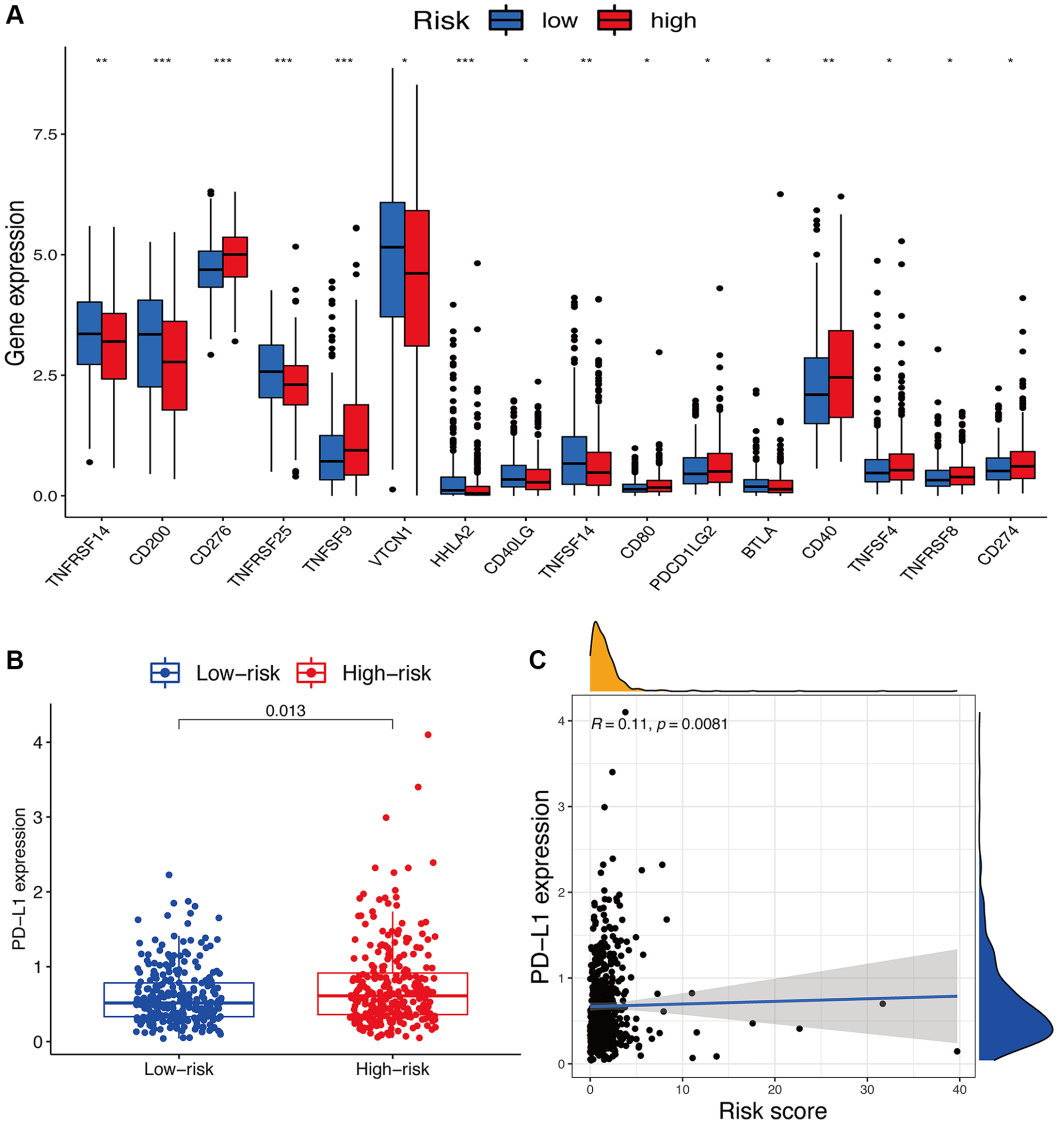
**Potential role of risk signature in immune checkpoints.** (**A**) Expression of immune checkpoints among two risk subgroups in UCEC patients. (**B**) Expression levels of genes PD-L1 in risk subgroups. (**C**) Correlation analysis between risk score and PD-L1.

For immune checkpoints, various identified immune-associated genes were significantly differentially expressed in the two risk subgroups (*p* < 0.05) (Figure 9A). Meanwhile, the patients with high-risk scores exhibited a dramatically low expression of several genes, including *TNFRSF14*, *CD200*, *TNFRSF25*, *VTCN1*, *HHLA2*, *CD40LG*, *TNFSF14*, and *BTLA*, but except for *CD276*, *TNFSF9*, *CD80*, *PDCD1LG2*, *CD40*, *TNFSF4*, *TNFRSF8*, and *CD274*. Moreover, we comprehensively analyzed the connection of *PD-L1* loci to the risk signature. We observed a dramatically increased *PD-L1* (Figure 9B) expression in high-risk score patients compared to low-risk score patients. Meanwhile, an obvious positive correlation was also found between *PD-L1* (Figure 9C) expression with the calculated risk score.

**Figure 10:**
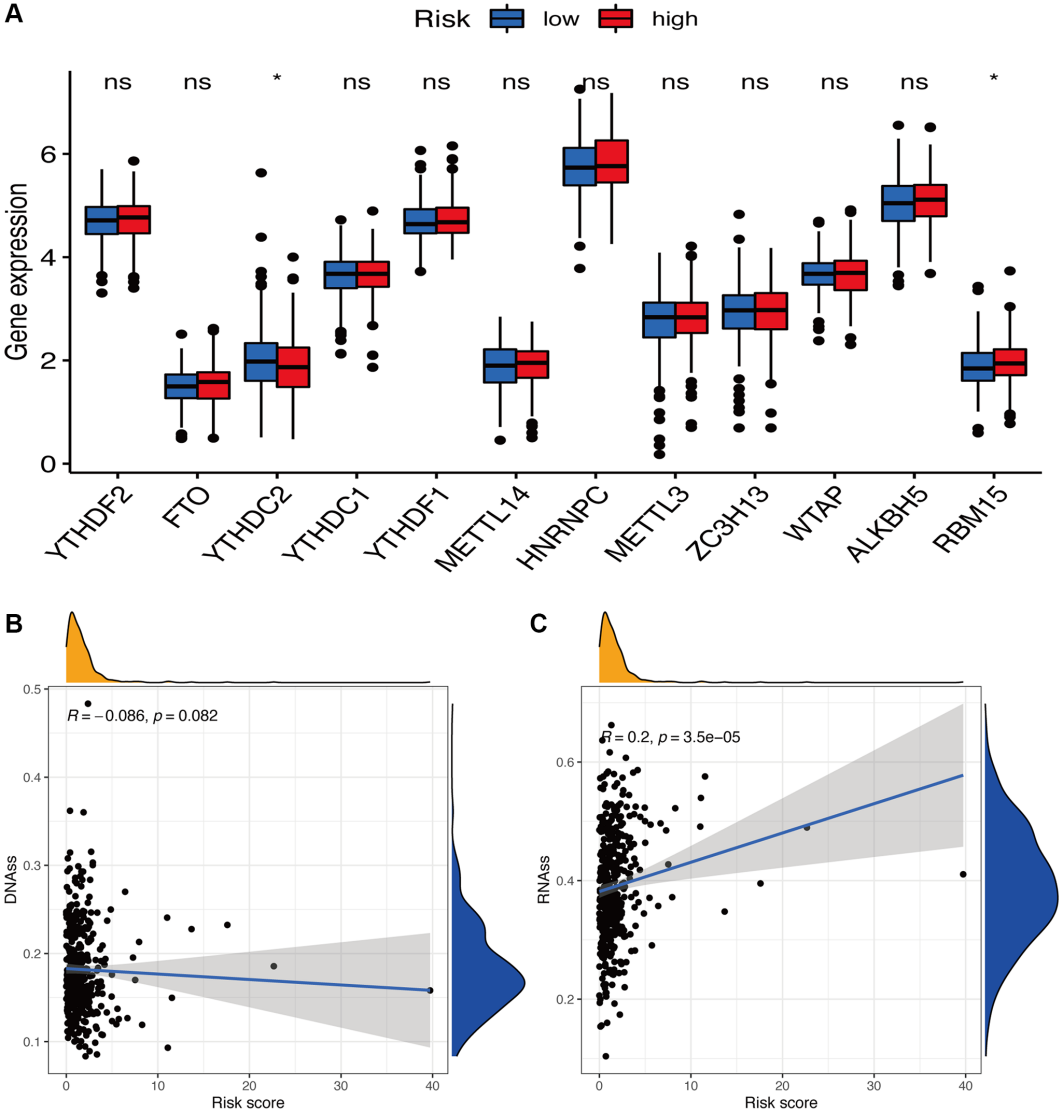
**Potential role of risk signature in m6A-related genes and tumor stemness.** (**A**) Expression of m6A-related genes among two risk subgroups in UCEC patients. Associations between risk signature and DNAss (**B**) and RNAss (**C**).

Tumor stemness, such as DNA methylation pattern and RNA stemness score, and m6A-associated genes are also key regulators of tumor progression. Compared to the subgroup with low-risk scores, the subgroup with high-risk scores exhibited dramatically decreased *YTHDC2* expression and increased *RBM15* expression (*p* < 0.05) (Figure 10A). Considering tumor stemness, a significantly positive correlation of the risk signature was observed with RNA methylation patterns (RNAss; *p* < 0.05) but not with DNA methylation patterns (DNAss; *p* > 0.05) (Figure 10B, 10C).

### The role of lncRNA HOXB-AS3 in UCEC cells

**Figure 11:**
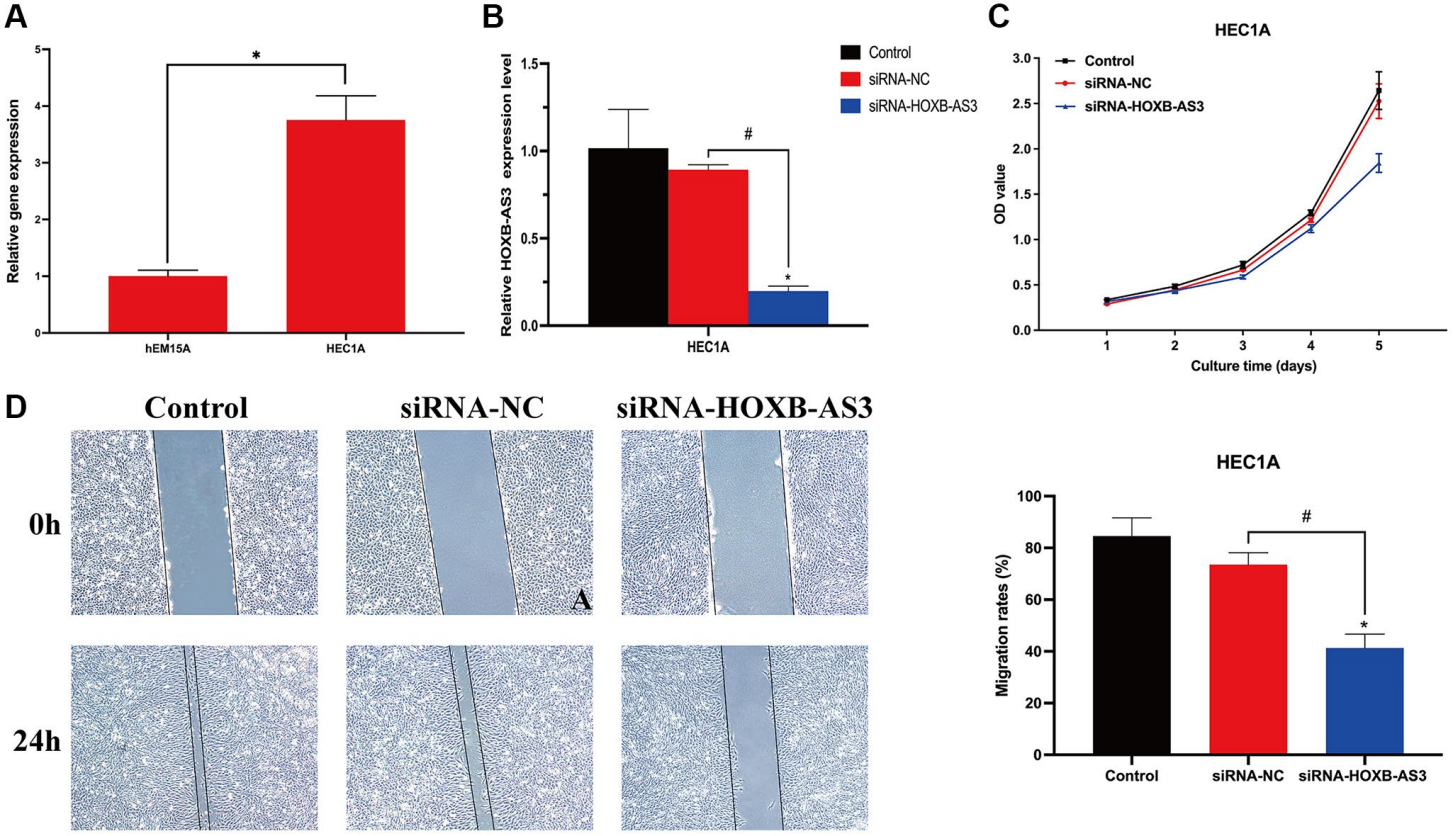
**Role of lncRNA HOXB-AS3 in UCEC cells.** (**A**) QRT-PCR analysis of SNCG. (**B**) The expression level of HOXB-AS3 in HOXB-AS3 inhibition model. (**C**) MTT assay. (**D**) Wound-healing assay.

Since the sequence of identified lncRNAs *AC009097.2*, *AL359220.1*, *AC100861.1*, *AC245884.9* has not been clarified in NCBI database, we finally validated the expression level of *HOXB-AS3* in HEC1A cell line. The lncRNA *HOXB-AS3* was significantly higher expressed in HEC1A cells than in hEM15A cells (*p* < 0.05; Figure 11A), which was completely consistent with the bioinformatic analysis.

To investigate the biological function of *HOXB-AS3* in UCEC cells, the model of *HOXB-AS3* inhibition was achieved by transfection of siRNA-HOXB-AS3 into HEC1A. As shown in Figure 11B, the expression of *HOXB-AS3* was dramatically reduced by siRNA-HOXB-AS3 (*p* < 0.05), and no significant difference was observed between control and siRNA-NC subgroups (*p* > 0.05). MTT assay revealed that the proliferative ability of HEC1A was distinctly hampered by *HOXB-AS3* deficiency as compared with that in victor transfected cells (*p* < 0.05; Figure 11C). Cell migration are another important aspect of cancer progression. Wound-healing assay results showed that HEC1A with *HOXB-AS3* depletion exhibited significantly lower scratch healing rate than those in siRNA-NC and control subgroups (*p* < 0.05; Figure 11D).

## DISCUSSION

Along with the development and application of next-generation sequencing technology in biological research, increasing biomarkers have been found for UCEC. However, biomarkers that can be used for early detection and prognostic prediction in UCEC are also urgently needed. As a newly identified mechanism for cell death, oxeiptosis plays an important role in the death of cancer [11]. In contrast, the role of oxeiptosis in the generation, development, progression, and metastasis of cancer is completely unclear. Moreover, lncRNA signatures related to oxeiptosis have also not been investigated. Herein, we identified and constructed a new risk signature and validated its high accuracy for the OS prediction of UCEC. Meanwhile, significant associations of this risk signature with tumor stemness, m6A-related genes, immune components, tumor microenvironment, and immune status were observed, suggesting its advantage.

Herein, to identify the relationships of lncRNAs with the OS of UCEC patients, we systematically analyzed oxeiptosis-related genes, including *PGAM5*, *KEAP1*, *AIFM1*, *NRF2*, and *AIRE*. Subsequently, for constructing a risk signature for the prediction of UCEC prognosis, five hub lncRNAs were employed: *AC009097.2*, *AL359220.1*, *AC100861.1*, *AC245884.9*, and *HOXB-AS3*. To verify the value of this constructed risk signature for the prediction of UCEC prognosis, many approaches were applied. We observed a close association of this risk signature with the tumor TNM stage, and grade. The American Joint Committee on Cancer (AJCC) staging system is commonly employed as a clinicopathological parameter [16]. Compared to the TNM stage, our risk signature could predict cancer grade, and prognosis of UCEC with high accuracy. Besides, the effectiveness of this risk signature for UCEC outcome prediction was also confirmed by a nomogram analysis.

Furthermore, obvious enrichments of hub lncRNAs were observed in immune-associated pathways, such as primary immunodeficiency, natural killer cell-mediated cytotoxicity, antigen processing and presentation, and immune network for IgA production. Meanwhile, significant connections of these lncRNAs to several immune cell infiltration were also found. Therefore, this association with immune processes indicated its predictive prognosis value. Interestingly, in the patients with high-risk scores, various immune cells exhibited significantly improved infiltration and immune functions. Due to the important roles of immune cells in anti-tumor immunity [17], we believe that the anti-tumor immune responses in UCEC patients with high-risk scores are dramatically proved. Additionally, we also discovered a close connection between the increased risk scores and the C3 subtype, suggesting its predictive value for OS and its protective value for UCEC.

Currently, by targeting immune checkpoints, immunotherapies are considered one of the most effective therapeutic methods to improve the outcomes of cancers [18]. The immune response of immunotherapies is significantly determined by *PD-L1* [19]. By blocking the *PD-L1*-mediated inhibition and enhancing T-cell functions, monoclonal antibodies against *PD-1/PD-L1* exhibited impressive therapeutic effects in clinical trials [20, 21]. Herein, we also verified *PD-L1* expressions in the patients from different subgroups and observed a positive correlation between their expressions and risk scores. Additionally, compared to the patients with low-risk scores, the patients with high-risk scores exhibited significantly differential expression of several immune checkpoint molecules, indicating the changes in immune responses in the patients in the high-risk group. Therefore, due to the predictive effect of our constructed risk signature on immune checkpoint expressions in UCEC patients, it can be used as a guideline for the immunotherapy of UCEC. However, the relationships of these oxeiptosis-related lncRNAs with immune-related genes also need further investigation.

Because of the invasive and self-renew activities, cancer stem cell-like cells (CSCs) can significantly promote tumor growth and progression. Herein, we observed a significant positive association of the lncRNA signature with the stem cell score, suggesting the role of this lncRNA signature as a UCEC risk factor.

As one of the most abundant methylations, m6A mainly occurs on the adenine of the RRACH sequence. Meanwhile, the participation of m6A in many human physiologies and cancers has been observed [22], especially in anti-tumor immune responses [23, 24]. Thus, clarifying the association of oxeiptosis with m6A is important. Herein, we could predict the expression of m6A-related genes, such as *YTHDC2* and *RBM15*. However, the underlying mechanisms for this connection remained uncovered.

Although this study identified hub oxeiptosis-associated lncRNAs in UCEC and proposed a risk signature that displayed a powerful prognostic value in UCEC patients, it still had some limitations. First, all gene expression and clinical UCEC data were obtained from public websites, and our conclusions should be validated by independent cohorts. Second, other prospective studies should be done to confirm further the results obtained from our current retrospective study. Additionally, functional and mechanistic studies are also needed to clarify the detailed function and mechanisms of oxeiptosis-associated lncRNAs in the progression of UCEC.

In summary, our study provided insights into the role of hub oxeiptosis-associated lncRNAs and developed a novel risk signature for UCEC patients. All identified lncRNAs could improve the prediction of overall UCEC survival and reflect patients’ immune conditions. This study was the first oxeiptosis-associated lncRNA signature for cancer, providing a novel perspective for therapeutic improvements in UCEC patients.

## MATERIALS AND METHODS

### Raw data acquisition

The normal endometrial cases and UCEC RNA sequencing datasets TCGA-UCEC (UCEC samples = 554, normal samples = 35) with reliable sources were collected from The Cancer Genome Atlas (TCGA, https://portal.gdc.cancer.gov/) database. Samples were obtained from Homo sapiens. According to previous studies [11], five oxeiptosis-associated genes (*PGAM5*, *KEAP1*, *AIFM1*, *NRF2*, and *AIRE*) were screened out and applied for further analysis. All public databases in this study were searched following relevant guidelines, and no ethical approval was required from the Ethics Committee of the First People’s Hospital of Linping District.

### Construction of the lncRNA Signature for prognosis

After evaluation of the connections between UCEC and oxeiptosis-associated lncRNAs and Pearson correlation analysis (|R^2^| > 0.2, *p* < 0.05), the “limma” R package was applied to identify differentially expressed lncRNAs related to oxeiptosis. Candidate lncRNAs were defined as the false discovery rate (FDR) < 0.05 and |log_2_ fold change (FC)| > 1 between tumor and normal tissues. Then, the “survival” R package was applied, and the univariate Cox regression analysis was performed to select the prognostic oxeiptosis-associated lncRNAs from all lncRNAs with a cutoff *p* < 0.01. We selected the overlapping lncRNAs between lncRNAs related to prognosis and differentially expressed as our candidate oxeiptosis-related lncRNAs. The “VennDiagram” package was applied to visualize the results in a Venn diagram.

After that, to identify the hub lncRNA and generate the lncRNA risk signature, we integrated these selected lncRNAs into a Lasso penalized Cox regression analysis. The risk score of the hub oxeiptosis-associated lncRNA risk signature was constructed using the following formula:


risk score=Σexplnci×βi

where explnci represents the relative expression of hub oxeiptosis-associated lncRNA i, and β is the regression coefficient. Then, UCEC patients were separated into low- and high-risk subgroups according to the median value of the constructed risk score.

### Predictive value of the lncRNA signature

For investigating the distribution of these two risk subgroups, the “ggplot2” and “Rtsne” packages were employed. Based on the levels of the risk scores, the prognostic ability was compared using Cox regression and survival analyses. Then, the “timeROC” package was applied to calculate the accuracy of this risk signature for prediction. For predicting UCEC patients’ outcomes, we constructed a nomogram using the “rms” package according to the risk scores, and the decision curve analysis was conducted to evaluate the accuracy and discrimination.

### Gene set enrichment analysis (GSEA) and gene set variation analysis (GSVA)

GSEA was applied to the gene expression matrix using the Hallmark and C7 gene sets v7.4. Enriched gene sets were used to detect KEGG pathways. Gene sets with *p*.adjust < 0.05 were considered significantly enriched after 1000 substitutions. GSVA was performed for each gene set and scoring. According to the GSVA score matrix, the changes at the gene-level were converted into changes at the pathway-level by the R package “GSVA”, and the potential biological functions were ultimately evaluated.

### Immune and stem cell-like features and m6A correlation analysis

The relationship between the expression of hub lncRNAs and immune cells infiltration was evaluated by CIBERSORT analysis. For exploring the immune functions and comparing the infiltration of immune cells between the two subgroups, a single-sample GSEA was performed. The association of the risk score with immune infiltration subtypes was tested by two-way analysis of variance (ANOVA). The relationship of the risk signature with the immune-associated genes was determined using the potential immune checkpoints retrieved from a previous study [25]. Next, we evaluated the associations of the risk signature with *PD-L1*. The relationships among m6A-related genes, tumor stemness, and risk score were assessed using Spearman correlation analyses.

### Cell culture

The human endometriosis cell line, hEM15A, and UCEC cell line, HEC1A, were purchased from American Type Culture Collection (ATCC) and cultured in Dulbecco’s Modified Eagle’s medium (DMEM; Gibco, Thermo Fisher Scientific, Waltham, MA, USA). The 10% FBS- (Hyclone, USA) and 1% penicillin/streptomycin- (Solarbio, China) contained medium was used for cell culture at 37°C under 5% CO_2_.

### Cell transfection

The short interference RNA that targets *HOXB-AS3* (si-HOXB-AS3; 5′-UGCUUGUCUGGAGAUGGAGCCACUA-3′) were synthesized by GenePharma Corporation (Shanghai, China), and HEC1A cell lines were divided into three subgroups: siRNA-HOXB-AS3 (transfected with siRNA-HOXB-AS3), siRNA-NC (transfected with nonspecific scrambled), and control (cells without transfection). Cells were performed with Metafectene transfection reagent (Invitrogen, USA) according to the manufacturer’s protocols. Briefly, siRNA was formulated with Metafectene transfection reagent and added directly to the cells after diluted into culture medium. The transfection efficacy was confirmed by qRT-PCR analysis.

### Cell proliferation analysis

Cells were seeded in 96-well plates at a density of 3500 cells/well. After 1 to 5 days of culture, cell viability was then assessed through MTT (3-(4,5-Dimethylthiazol-2-yl)-2,5-diphenyltetrazolium bromide) analysis, and the absorbance was assessed at 490 nm.

### Wound-healing assay

Cells were seeded in a 6-well plate and incubated for 24 h. A wound was created by the sterile 200 μL pipette tip at cell surface. The wound closure was quantified at 0 h and 24 h after the wound was created using Image J software (NIH, Bethesda, MA, USA).

### qRT-PCR analysis

Trizol reagent (Invitrogen, USA) was used to isolate RNA from cell lines. The RNeasy mini kit (QIAGEN, USA) was used to isolate RNA. Then, the RNA was reverse transcribed using a Transcriptor First-strand cDNA synthesis kit (Roche, Switzerland). SYBR green master mix was used to perform qRT-PCR using an Applied Biosystems 7500 Real-Time Cycler (Applied Biosystems, USA). Gene expression was measured using the 2^−ΔΔCT^ method. *GAPDH* (Forward: CTGCCTCGATGGGTGGAGTC; Reverse: GAGTTAAAAGCAGCCCTGGTG) was used as a normalization control. Primer sequences for *HOXB-AS3* as follows: Forward: TGCTTGTCTGGAGATGGAGC; Reverse: GATAAGAGCGATGAGGCGCT.

### Availability of data and materials

The datasets used and/or analyzed during the current study are available from the corresponding author on reasonable request.

## Supplementary Materials

Supplementary Table 1

Supplementary Table 2

Supplementary Tables 3 and 4
