# Supplementary material for: Identification and validation of oxeiptosis-associated lncRNAs and prognosis-related signature genes to predict the immune status in uterine corpus endometrial carcinoma
Source: Aging (Albany NY). 2023 May 19;15(10):4236–52. doi: 10.18632/aging.204726 (PMC10258008; doi:10.18632/aging.204726)
Supplement: Supplementary Tables 3 and 4 [file aging-15-204726-s003.pdf]

SUPPLEMENTARY TABLES

Supplementary Table 3. Prognosis-associated lncRNAs in UCEC.

lncRNA	HR	HR.95L	HR.95H	*P*-value
HOXB-AS3	0.93628118	0.89150989	0.98330086	0.00844932
AC009097.2	0.27067064	0.11076392	0.66143018	0.00414801
AC006230.1	0.05786056	0.00716807	0.46704964	0.00748491
AP001107.9	1.98546001	1.20101534	3.28226568	0.00749225
AL359091.3	1.14538382	1.04438971	1.25614421	0.00394941
BOLA3-AS1	1.53162029	1.25310438	1.87203935	3.14E-05
AC007786.1	2.08596778	1.20413416	3.61360198	0.00872689
AL928654.1	1.55138934	1.16457958	2.06667617	0.00268907
AC005393.1	1.16390935	1.03873094	1.30417313	0.00893539
AL359220.1	0.04652139	0.00495003	0.43721763	0.00728142
AC078883.1	0.12197381	0.03199084	0.46505842	0.0020621
AL590369.1	1.28693067	1.08644386	1.52441429	0.00350576
LINC00618	3.14800926	1.37601403	7.20193404	0.00660945
VIM-AS1	0.66771828	0.51120849	0.87214456	0.00303829
AC100861.1	2.13325286	1.3666027	3.32998593	0.00085423
AC019131.2	0.50135844	0.3087966	0.81399952	0.00523449
AC026202.2	0.21468155	0.07106356	0.6485485	0.0063794
AC245884.9	1.60864576	1.12933399	2.29138698	0.00844301
AC003102.1	0.630108	0.45478537	0.87301862	0.00549914
ZDHHC20-IT1	1.21793691	1.05742258	1.40281694	0.00625114
AL078587.2	1.26314258	1.05879642	1.50692724	0.00947259
AC002467.1	2.88591679	1.4147046	5.88710585	0.00357152

Supplementary Table 4. Five hub lncRNAs were identified by lasso penalized Cox regression analysis.

LncRNA	Coef	HR	HR.95L	HR.95H	*P*-value
HOXB-AS3	−0.0475656	0.95354795	0.90926026	0.99999278	0.04996521
AC009097.2	−1.0073937	0.36516947	0.1486939	0.89680037	0.02797969
AL359220.1	−2.1630737	0.11497119	0.01047333	1.26209829	0.07680413
AC100861.1	0.76949971	2.15868601	1.3281459	3.50859442	0.00190221
AC245884.9	0.46785307	1.59656281	1.14870431	2.21903302	0.00534767
